# A New Method for a Piezoelectric Energy Harvesting System Using a Backtracking Search Algorithm-Based PI Voltage Controller

**DOI:** 10.3390/mi7100171

**Published:** 2016-09-23

**Authors:** Mahidur R. Sarker, Azah Mohamed, Ramizi Mohamed

**Affiliations:** Department of Electrical, Electronic and System Engineering, Faculty of Engineering and Built Environment, Universiti Kebangsaan Malaysia (UKM), Bangi 43600, Malaysia; azah_mohamed@ukm.edu.my (A.M.); ramizi@ukm.edu.my (R.M.)

**Keywords:** energy harvesting, optimization, micro devices, low voltage, controller

## Abstract

This paper presents a new method for a vibration-based piezoelectric energy harvesting system using a backtracking search algorithm (BSA)-based proportional-integral (PI) voltage controller. This technique eliminates the exhaustive conventional trial-and-error procedure for obtaining optimized parameter values of proportional gain (Kp), and integral gain (Ki) for PI voltage controllers. The generated estimate values of Kp and Ki are executed in the PI voltage controller that is developed through the BSA optimization technique. In this study, mean absolute error (MAE) is used as an objective function to minimize output error for a piezoelectric energy harvesting system (PEHS). The model for the PEHS is designed and analyzed using the BSA optimization technique. The BSA-based PI voltage controller of the PEHS produces a significant improvement in minimizing the output error of the converter and a robust, regulated pulse-width modulation (PWM) signal to convert a MOSFET switch, with the best response in terms of rise time and settling time under various load conditions.

## 1. Introduction

**Figure 1:**
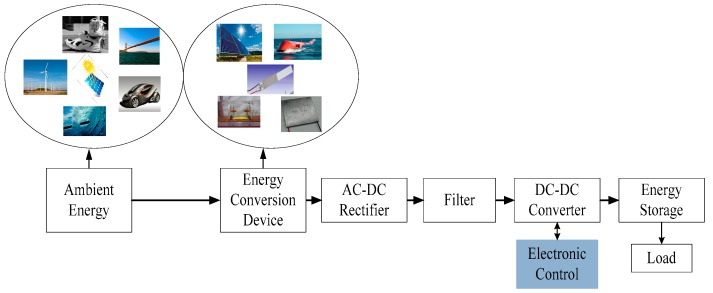
Block diagram of vibration energy harvesting system.

The energy harvesting from the ambient energy sources, such as solar, wind, thermal, sound, vibration, solid waste, etc., are commonly popular now-a-days because of rising power demand [1,2,3,4]. Along with the renewable energy generation methods, micro-level energy harvesting has become the focus of new researchers and developers to power ultra-low power devices at remote or hard-to-reach zones. There are three techniques to harvest energy from vibration, including electrostatic, electromagnetic, and piezoelectric. Among them, harvesting the micro-level energy utilizing piezoelectric vibration transducers (PVT) is now a greater concern to be researched [5,6,7,8]. The block diagram of a piezoelectric energy harvesting system (PEHS) is shown in Figure 1; it can be summarized into three core components: piezoelectric devices, the power electronic interface, and electrical energy storage.

One of the most popular power electronic converters for energy harvesting applications is a single-direction DC-DC converter. The DC-DC converters are called boost, buck, and buck-boost. The principle of such a converter is that the output voltage magnitude increases or decreases compared to the input voltage magnitude from ambient sources [9,10,11,12,13]. Operated under an open loop condition, the DC-DC converter displays poor voltage regulation and insufficient active response, and such converter is used as a closed loop feedback control system for output voltage regulation. Some researchers have proposed to switch the converter on and off (duty cycle) to achieve the optimal output voltage from the controller. Power flow is regulated through high frequency pulse width modulation (PWM) for switching control of the MOSFETs in the circuitry of the DC-DC converter [14,15,16]. The advantages of the single-phase DC-DC converters is the need of a single MOSFET to be switched, since its losses are small and is suitable for the energy harvesting applications.

Usually, the proportional-integral (PI) controller has been largely utilized in controlling various applications due to its normal control, simple design and low-cost maintenance, robustness, and ruggedness [17]. Additionally, the PI controller is utilized to regulate voltage, current, etc. Hence, the advantage is to utilize a PI controller to solve different technical problems, such as high overshoot, high steady state error, and torque, due to changes in mechanical load [18]. In [19], a conventional PI controller is proposed to control a boost converter. On the other hand, the disadvantage of a PI controller is the requirement of mathematical modeling and the trial-and-error technique [20,21]. The difficult part is to find the best parameter values, such as the proportional gain (Kp) and integral gain (Ki). Generally, those parameters of the controller need manual tuning to obtain the best values, and is time consuming. There are some known techniques to find the optimal PI voltage controller values, such as the Ziegler–Nichols method, Cohen-Coon method, and Lambda tuning method, etc. However, such techniques have certain disadvantages, such as being trial-and-error methods, require manual tuning, and complex mathematical models [18,22]. Some optimization algorithms, such as genetic algorithm proportional-integral (GA-PI) optimization [23], bacterial foraging proportional-integral-derivative (BF-PID) optimization [24], and particle swarm optimization proportional-integral-derivative (PSO-PID) optimization [25], have been utilized to enhance the performances of PI controller and to obtain the parameters of a PI controller. The backtracking search algorithm (BSA) method is an optimization technique that has the merits of stability, toughness, and the ability for global convergence, yet is simple to implement [26].

In this study, the BSA optimization technique is developed for tuning the parameters of a PI controller for a PEHS. The results of the proposed methods are compared with the results of PSO-based PI controller. The proposed BSA-PI controller obtained the minimum objective function (MAE) faster than the other techniques in terms of rise time, settling time, and in all aspects. The rest of the paper is organized as follows: Section 2 describes the design of the PI voltage controller based on BSA for a PEHS; Section 3 presents the proposed BSA-PI voltage controller for a vibration-based PEHS; Section 4 presents a simulation model PI voltage controller for the PEHS utilizing the proposed BSA technique; Section 5 presents the experimental set-up; Section 6 presents the results and discussion; and, finally, the conclusion is given in Section 7.

## 2. Design PI Voltage Controller Based on BSA for PEHS

The traditional and popular PI controller, uses two loops for control. One is the inner current control loop and the other is the outer voltage control loop. BSA is a systematic method to iterate through all of the possible configurations of a search space algorithm developed by [27]. It is a general technique which finds the solutions of computation problems, notably constraint satisfaction problems for each individual application.

### 2.1. Conventional PI Voltage Controller

PI controllers are the most common single-input-single-output (SISO) feedback controllers in use in industry and others applications. The standard format of the controller is shown below in Equation (1) [28]:

(1) $$\mathrm{Controller}\;\mathrm{Output}=K_{p}e+K_{i}\int_{0}^{t}edt$$

where *e* is the error, *K_p_* is the proportional gain, *K_i_* is the integration gain factor, and *t* is the time. The performance of a PI controller is highly dependent on its gains. If these gains are chosen improperly, the output will experience overshoot, become oscillatory, or even diverge. The adjustment of these controller gains to the optimum values for a desired control response is called tuning of the control loop. There are different methods documented in the literature for the tuning of PI controllers, which may not necessarily result in the optimum gains of the controller. In addition, the most effective tuning methods are the use of different optimization techniques. In this study a BSA optimization-based PI voltage controller has been considered. 

### 2.2. Conventional Backtracking Search Algorithm (BSA)

**Figure 2:**
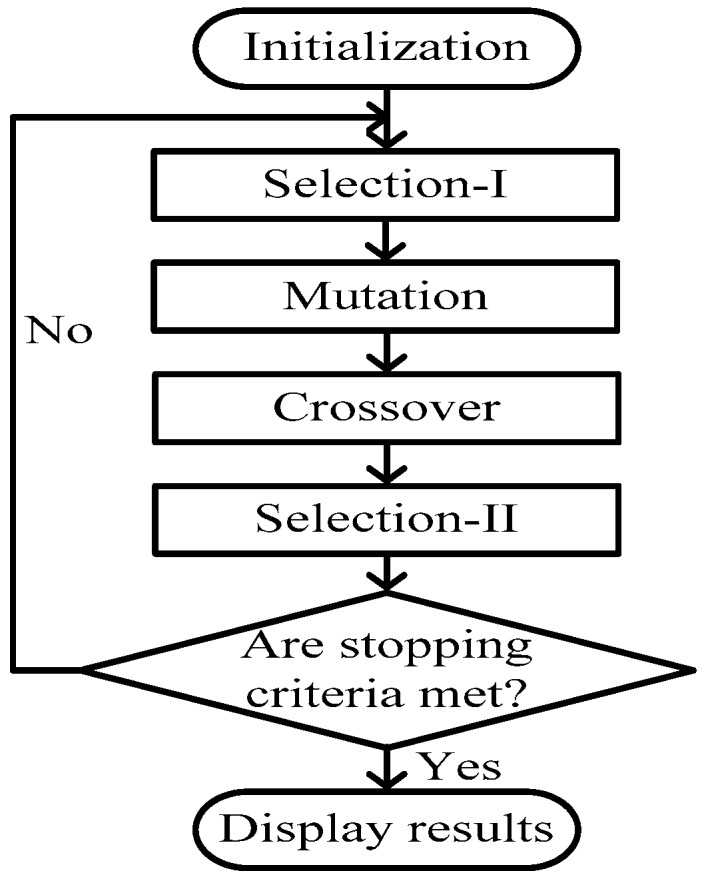
General flowchart of backtracking search algorithm (BSA).

BSA is a population-based iterative algorithm designed to be a global minimizer. This allows searches to be flexible and less time consuming for attempting to recreate the initial direction [27]. Usually, BSA is used to solve constraint satisfaction problems. The BSA is divided into five processes as shown in Figure 2.

### 2.3. Control Strategy

**Figure 3:**
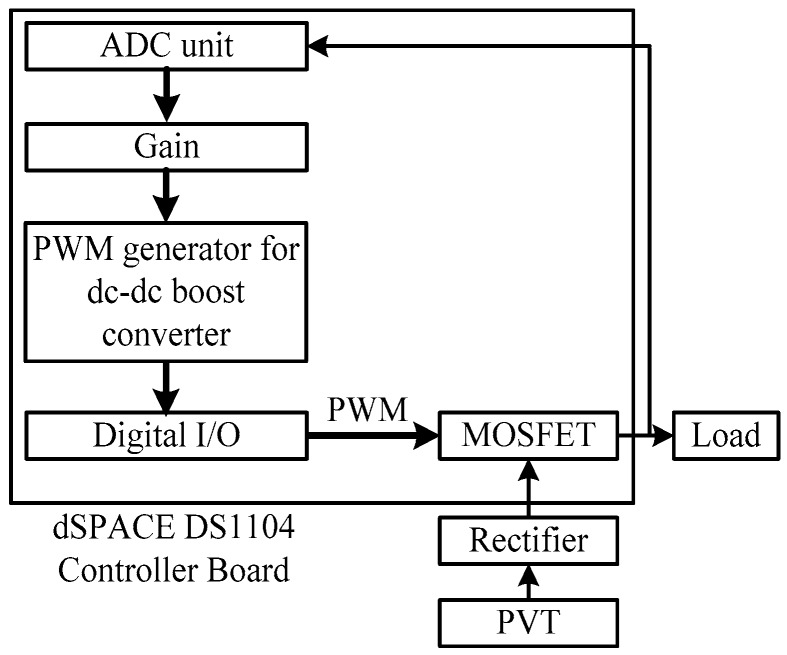
Block diagram of the dSPACE DS1104 controller converter.

The PI controller is an active controller to adjust the single-phase boost converter DC output voltage for a PEHS. The block diagram of a dSPACE controlled converter described for this study is shown in Figure 3, which includes a PVT, a rectifier with a filter, a dSPACE DS1104 controller (dSPACE GmbH, Paderborn, Germany), boost converter, and a load. The feedback loop is utilized for connecting output voltages of converter, with the DSPACE. The implementation of the MATLAB/Simulink (Mathworks Ltd., Cambridge, UK) converter control algorithm simulated in real-time is accomplished using a dSPACE DS1104 RTI. Additionally, the dSPACE input-output library block is required to include in the control algorithm [29,30].

The analog-to-digital converter (ADC) terminal of the dSPACE DS1104 is used for additional signal handling. With a standalone converter, the approach is to control the output voltage supplied to the load. The duty ratios of switching devices are modified by the control signal, which is the preferred necessary frequency of the converter output [31]. The triangular signals of 10 kHz to produce the switching signals conduct the MOSFET. The frequency of the triangular signal finds the converter switching frequency at which the MOSFET is switched.

## 3. Proposed BSA-PI Voltage Controller for Vibration-Based PEHS

The proposed BSA-PI voltage controller has gained popularity because of its easy design, low-cost for maintenance, and it does not depend on a mathematical model. However, the traditional PI controller has demerits, such as a required mathematical model and need for a trial-and-error technique to obtain the Kp and Ki values for developing the controller. 

### 3.1. Backtracking Search Algorithm (BSA)

Various optimization techniques have been modified to increase the system efficiency, such as PSO, GA, gravitational search algorithm (GSA), and ant colony search technique, are utilized to solve real-valued mathematical optimization problems. Usually, to solve difficult mathematical optimization problems, non-linear and non-differentiable optimization methods are utilized. BSA optimization is a metamorphic computation method established by [27]. The assumption of BSA for generating a sample population consists of two innovative edges and mutation operators. BSA governs the value of the find on the best populations and, in the location boundary, conducts a robust investigation for possibilities of abuse [27,32,33,34,35]. Thus, BSA optimization has been demonstrated in significant research as one of the most powerful optimization methods [33,34,35].

### 3.2. Optimal PI Voltage Controllers

The optimization technique combines three necessary features, including input information, an objective function, and optimization limitations. All elements are working for development and arrangement to achieve optimal PI parameter values. The optimization technique is to obtain the best solution by minimizing the objective function though the input data and the collection of the limitations in all generations of the iterative procedure.

### 3.3. Input Information

The input data for the PI voltage controller of the optimization technique are a number of boundary PI optimal parameter values for input and output. The input matrixes build a number of columns and rows. The numbers of columns are produced by the mathematical values to the boundary for error and alteration of error. The output of the optimal PI parameter values are the number of rows generated by the size of populations, as shown in the matrix:

(2) $$B_{ij}=\left[\begin{array}{cc} X_{ij} & X_{1j}\\ X_{i1} & X_{ij} \end{array}\right]$$

where *B* is the input data of the optimization technique, *i* = 1, 2, …, *A*, *A* is the size of the population, and *j* = 1, 2, …, *S*, where *S* is the problem dimension. In this study, two problem dimensions and a population of 50 have been considered to obtain the best parameter values for the PI voltage controller.

### 3.4. Objective Function

An objective function is essential for the optimization method to achieve a minimum error. Therefore, the objective function finds the best value of the PI voltage controller output to improve the system permanence. The MAE is utilized as an objective function to find appropriate parameter values for controller for best results. The MAE function can be calculated as shown in Equation (3).

(3) $$\mathrm{MAE}=\frac{1}{H}\sum_{i=1}^{H}\left|error\right|$$

Here, *H* is the number of sample, *error* is PI voltage control for boost converter.

### 3.5. BSA for an Optimal PI Voltage Controller

The BSA has been developed to choose some important parameters; for example, the number of iterations (*I*), the size of the population (*A*), and the number of problem variables (*S*) for the PI voltage controller. The BSA is divided into five processes, including initialization, selection-I, mutation, crossover and selection-II [32,33,34,35].

#### 3.5.1. Initialization

The initialization method is an archaic structure for the population of the mathematical values of the PI parameter (*T_ij_*) described by the following Equation (4):

(4) $$T_{ij}=arb.\left(up_{j}-low_{j}\right)+low_{j}$$

where *i* = 1, 2, 3, …, *A* and *j* = 1, 2, 3, …, *S*, here *A* is the population size and *S* is the problem dimension, respectively. The objective function (MAE) is calculated for each population [27,28,29,30,31].

#### 3.5.2. Selection-I

In the BSA’s Selection-I define the historical population (*oldT_ij_*) to be utilized for calculating the find direction. The primary historical population is defined utilizing Equation (5):

(5) $$oldT_{ij}=arb.\left(up_{j}-low_{j}\right)+low_{j}$$

The *oldT_ij_* remembers the population arbitrarily selected from the former propagation for building the find direction matrix, holding the biased benefit of earlier contract to produce a new trial population. The comparison between two arbitrary values is shown in the following condition:

if *a < b* then *oldT_ij_ = T_ij_* (6)

*oldT_ij_* = *permuting*(*oldT_ij_*) (7)

#### 3.5.3. Mutation

Mutation is a method that generates the new population of the primary and history population as shown in Equation (8), in which *arb.* is the standard normal distribution, the *E* value controls the amplitude of the search-direction matrix:

*Mutant* = *T_ij_* + *E.arb.*(*oldT_ij_* − *T_ij_*) (8)

BSA produces a sample population, and then accepts a partial benefit of its experiences from earlier propagation.

#### 3.5.4. Crossover

In this procedure, the sample of population *G_ij_* is created. The primary sample of the populations is occupied from mutation, as shown in Equation (8). The crossover contains of two parts. The first part creates the binary matrix called *map_ij_*, and the second part is procedure comparison between population *T_ij_* and trial population *G_ij_*. Crossover is utilized to achieve updates in *map_ij_*.

#### 3.5.5. Selection-II

**Figure 4:**
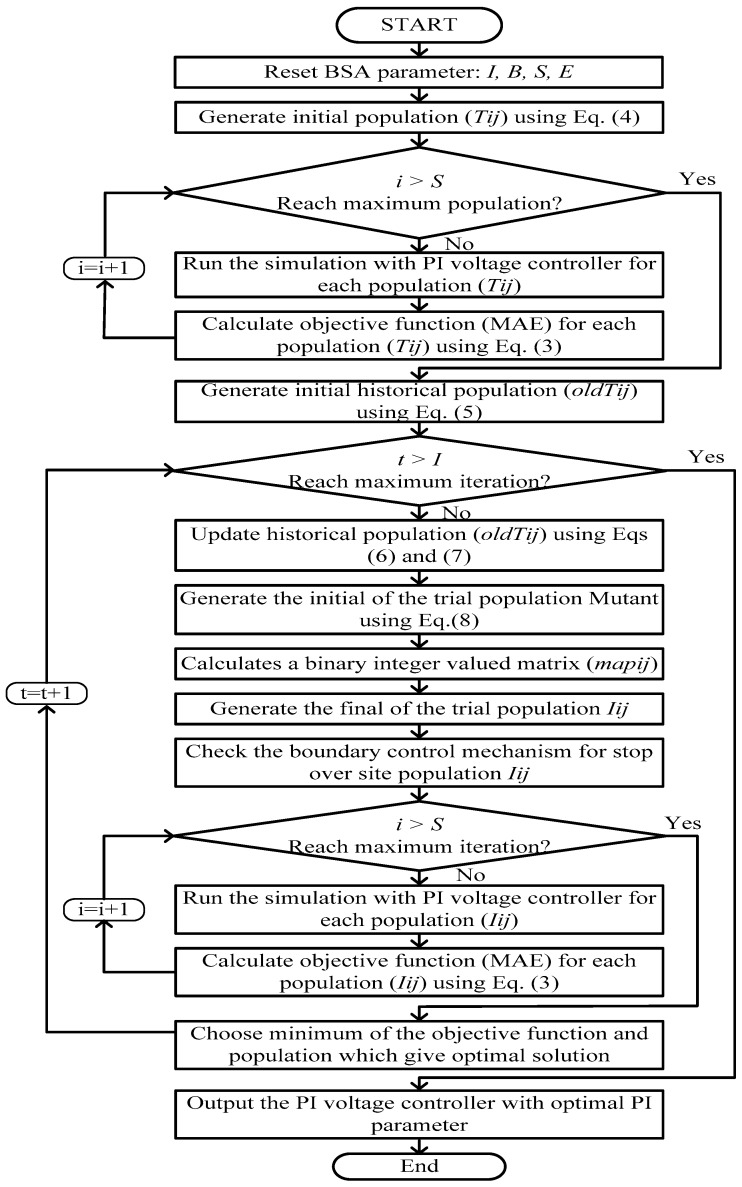
Proposed BSA-based optimum proportional-integral (PI) voltage controller design procedure.

Here, the optimization method runs to compare the population *T_ij_* and trial population *G_ij_* to achieve the best population and fitness value. Figure 4 shows the flow diagram explaining the BSA-based PI voltage controller.

## 4. Simulation Model PI Voltage Controller for PEHS Utilizing the Proposed BSA Technique

**Figure 5:**
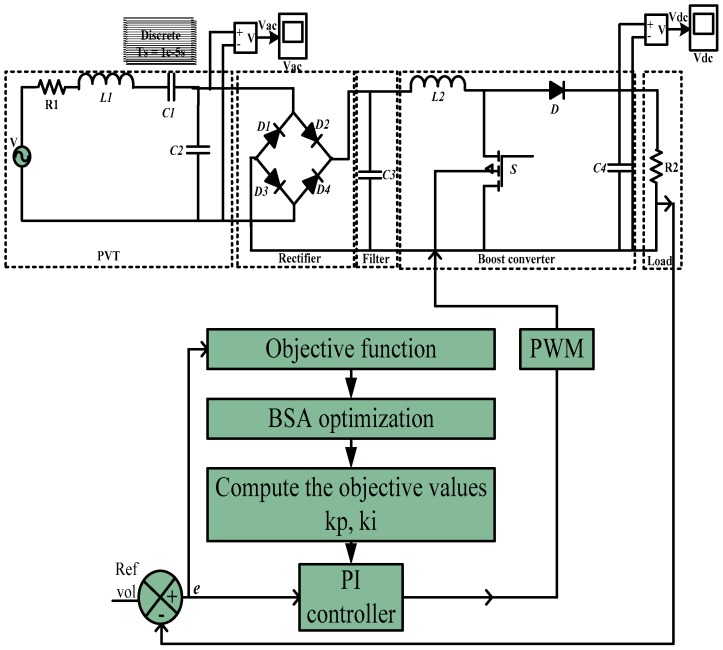
Block diagram of the proposed BSA-based PI voltage controller for piezoelectric energy harvesting system (PEHS).

The completed PI voltage controller simulation model for the PEHS is developed using the PVT (as an input source), full-wave rectifier, low pass filter, and boost converter with temporary storage devices as shown in Figure 5. This figure denotes the schematic diagram of the developed MATLAB/Simulink PEHS.

The functions of the integrated energy harvesting system circuit are as follows: the PVT performs as a bending mechanical structure that produces an amount of charge from force. The electrical equivalent circuit of the PVT is simulated using Matlab/Simulink software. The role of the PVT is to be stressed mechanically by a force vibration, when one side of PVT is flexed, then the other side is free to move. When the PVT vibrates, its electrodes receive an amount of charge that tends to counteract the imposed strain. This amount of charge is harvested, stored, and delivered to power electrical circuits. The PVT is interfaced with a power electronic network that extracts power and delivers it to a storage device, such as a rechargeable battery or a supercapacitor. Since the piezoelectric crystal-based PVT generates low AC voltage output with fluctuations and harmonics, it is difficult to process this low level signal at various magnitudes. At the end, the AC power that is harvested from an energy-harvesting PVT needs to be rectified through a standard diode bridge before it can be delivered to storage. The output of the rectifier DC voltage contains ripples. Usually, these ripples are at a maximum for a half-wave rectifier and a minimum for a full-wave rectifier. For most of the applications, direct voltage from a rectifier to the load leads to poor operation of the circuit. If the rectifier output is smooth and steady, then the overall operation of the circuit improves because the filter circuit reduces the ripples and produces a constant DC voltage. However, the theoretical rate of power extraction can be greatly improved by interfacing the harvesting transducer with a power electronic switching converter. Power electronic converters are capable of imposing static or dynamic relationships between the voltages and currents of the PVT by operating the transistors like switches. One of the most popular power electronic converters for energy harvesting applications is the single-phase DC-DC converter. This converter has become the subject of considerable interest for low-power PEHS applications. 

**Figure 6:**
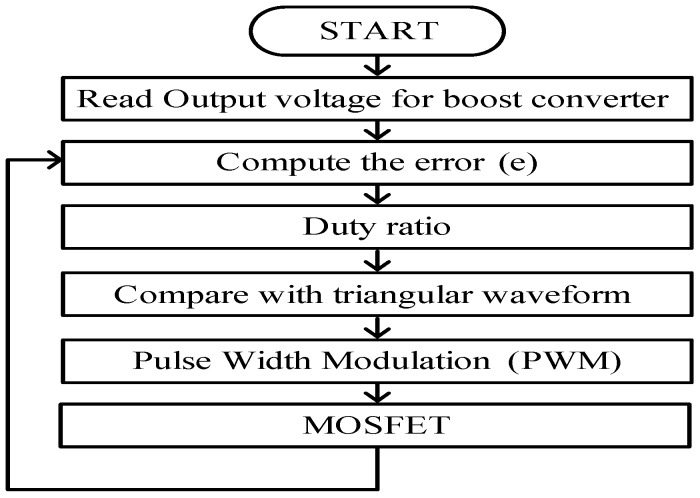
Flowchart of the control algorithm for the PEHS.

The PWM switching technique is used for controlling the PEHS by the BSA method. The PEHS model is surveyed depending on the voltage feedback signal. All possible cases of voltage drops have been considered during the change in loads in the simulation model by constructing a very powerful controller for the PEHS. The developed controller is used to obtain the best values for the PI voltage control parameters by the BSA optimization technique. The PWM technique receives Kp and Ki values from the PI controller and generates switching signals for the boost converter of the MOSFET for gate drives to deliver DC voltages to the PEHS. However, the BSA-PI voltage controller is used to obtain suitable PI parameter values. Figure 6 shows flowchart of the control algorithm for PEHS.

## 5. Experimental Setup

The hardware structure is the depiction of an electronic system, method, and to regulate implementation of the design for such a prototype system. The hardware implementation is one of the vital parts to verify the circuit functionality is in proper operation.

**Figure 7:**
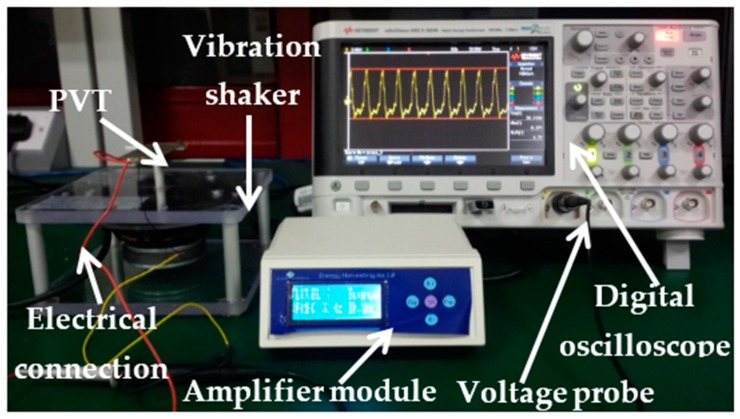
Experimental test bench layout.

The PVT bender is a pre-mounted and prewired double quick-mount bending generator. The use of a frequency generator with a power amplifier is preferred to control the mechanical energy generation by varying the frequency. The experimental test setup for data extraction from the PVT is shown in Figure 7. To conduct the PVT analysis, the natural frequency has been changed manually between 12 Hz and 74 Hz through the amplifier unit, investigating the resonance frequency of the PVT details as shown in the Results and Discussion (Section 6).

### 5.1. Piezoelectric Vibration Transducer

**Figure 8:**
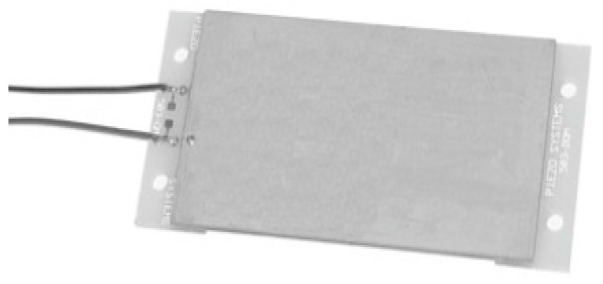
Prototype of the piezoelectric vibration transducers (PVT).

A single PVT has been tested with the energy harvesting power conversion circuits. The prototype of a PVT is shown in Figure 8. The parameter values of the PVT are summarized as shown in Table 1. The energy harvesting PVT is a pre-mounted and prewired double quick-mount bending generator designed to attach easily to sources of mechanical strain.

### 5.2. Experimental Setup of the PEHS

**Figure 9:**
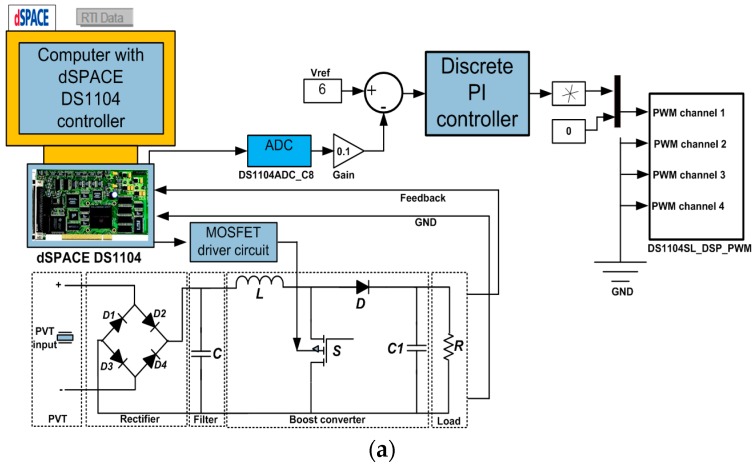

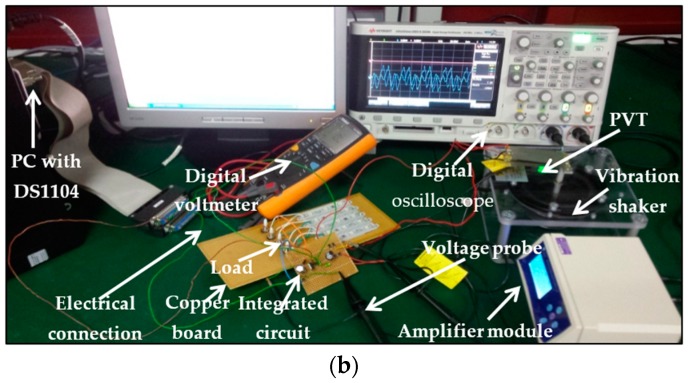
(**a**) dSPACE-based PEHS prototype implementation block diagram; and (**b**) in-lab PEHS prototype experimental setup.

As a part of the prototype development of the PEHS, the performance evaluation is essential to verify the conformance with the simulation. To verify the simulated model, an integrated prototype for the PEHS was constructed, tested, and evaluated in the laboratory, which is shown in Figure 9a,b. This equipment is considered to build a prototype system for the PEHS as shown in Table 2. To verify the simulation results, a hardware prototype for the PEHS was fabricated in the laboratory.

## 6. Results and Discussion

This section describes the results of the PVT and optimal results of the BSA-PI and the PSO-PI voltage controller algorithms of the PEHS. 

### 6.1. Extracting Data from the PVT

**Figure 10:**
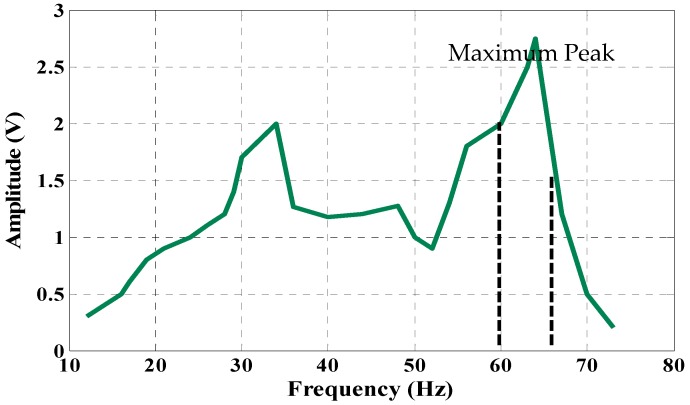
Frequency spectrum of the PVT.

This experiment was conducted to obtain the behavior of the PVT by using energy harvesting kits, an amplifier module, and shaker. The amplifier module is set to vary of the input frequency manually to generate the vibration signal at different levels and different frequency ranges to obtain the response of the PVT. The analytical output of the PVT is shown in Table 3. Figure 10 shows that the PVT produces maximum output at 65 Hz. The input of 65 Hz is used to develop the simulation and experimental models of the PEHS. 

### 6.2. Simulation and Analysis

**Figure 11:**
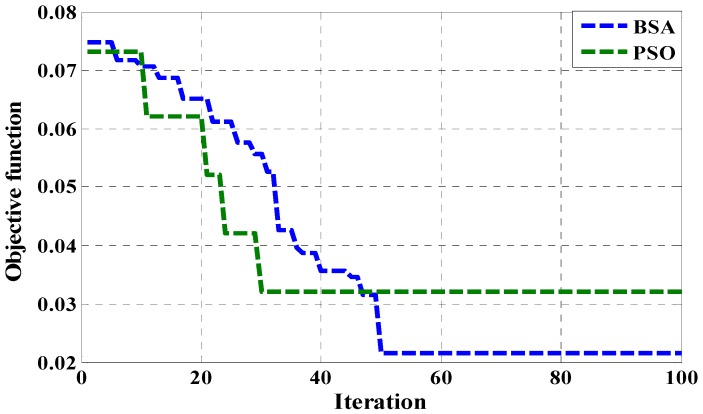
Performance comparison based on BSA and particle swarm optimization (PSO).

This section describes the outcome of the improvement results of the BSA optimization-based PI voltage controller for the PEHS. The simulation results have been compared with the hardware results to verify the functionality of the developed PEHS. The PI voltage controller parameters were effectively tuned by the BSA optimization technique to obtain the optimal (Kp, Ki) parameter values for the PI. This process has also been led through PSO optimization techniques to find the optimal PI parameter. Population-based optimization techniques can find optimal solutions for almost all types of problems. However the mathematical optimization technique cannot find any solution when the problem dimension is high. This comparison is used to estimate and define the robustness of the BSA optimization. Figure 11 represents the optimization performance of the BSA and PSO using the objective function of MAE for 100 iterations. The BSA optimization technique has obtained the minimum objective function MAE values. Table 4 shows the values of the output error by different techniques. It is observed in Table 4 that the BSA technique produces the minimum error in optimizing the parameter values of the PI voltage controller and regulates the PWM signal so that the duty of PWM output of the controller exhibits robustness, ruggedness, and efficiency. To ensure that the comparisons are fair, the same population size and maximum number of iterations that was applied in both techniques are shown in Table 5. 

### 6.3. Simulation and Experimental Results in Terms of Rise and Settling Time for the PEHS

**Figure 12:**
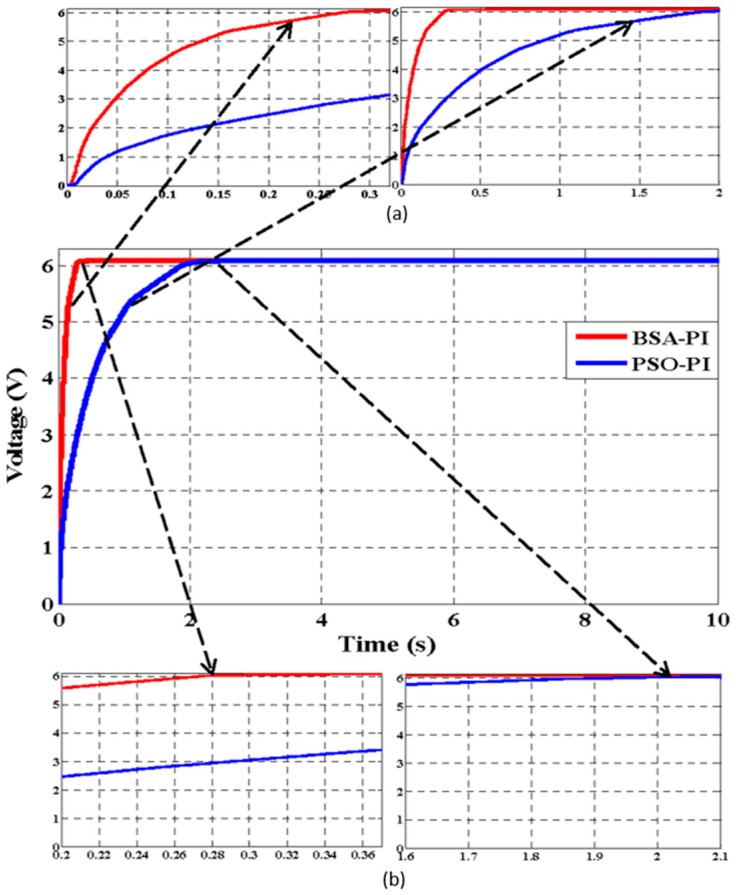
Simulation results BSA-PI and PSO-PI controller with respect to rise time (**a**) and settling time (**b**).

**Figure 13:**
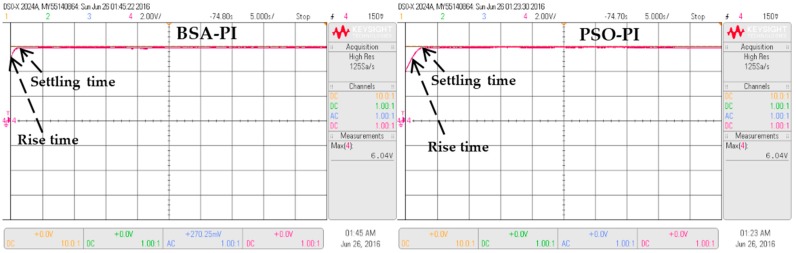
Experimental results PSO-PI and BSA-PI controller with respect to rise and settling time.

The objective function, as shown in Equation (3), is optimized by BSA-PI while satisfying the constraints in Equations (4)–(8). To validate the optimization results, the outcomes of BSA-PI are compared with PSO-PI. The responses of the PEHS simulation results using PSO-PI controllers and the BSA-PI controller are shown in Figure 12. From Figure 12 it can be seen that BSA-PI can find the best optimal result with respect to the rise time value of the BSA-PI controller is less time than the PSO-PI controller, and settling time is comparatively large for PSO-PI, but less for BSA-PI. The hardware results agree well with the simulated results shown in Figure 13. Table 6 represents the comparison of the optimized output using these techniques. The BSA-PI has successfully reached the best result compared with the other PSO-PI controller in terms of the rise time and settling time.

### 6.4. Simulation and Experimental Results of a Fully-Integrated PEHS

**Figure 14:**
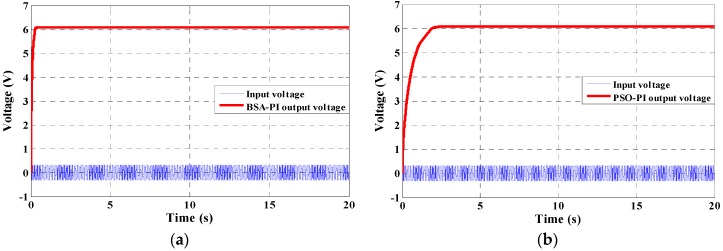
Simulation results of a complete PEHS utilizing a BSA-PI (**a**) and a PSO-PI (**b**) controller.

**Figure 15:**
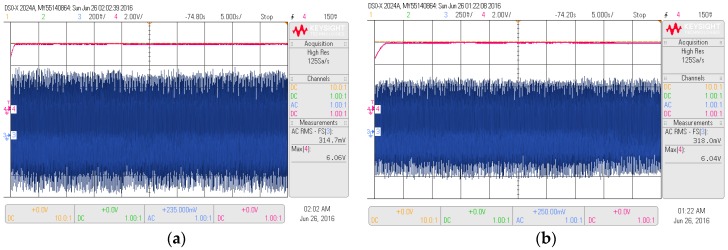
Experimental results of a complete PEHS utilizing a BSA-PI (**a**) and a PSO-PI (**b**) controller.

This study also analyzes the simulation and experimental results of a fully-integrated PEHS input and output voltage using the proposed new technique. The results using the BSA-PI and PSO-PI techniques are shown in Figure 14a,b. From Figure 14a it can be seen that the input sinusoidal AC Vrms 0.3 V generated from the PVT and boost harvested an optimal output voltage of 6.06 V DC utilizing the proposed BSA-PI technique of the PEHS. Initially the curve bends, but after 0.2 s, the curve becomes steady. On the other hand, from Figure 14b, it is shown that the voltage output characteristics of the PSO-PI technique are similar without any significant difference for a changing load. Initially the curve bends, but after 2.1 s, the curve becomes steady. The peak overshoot value is similar for the two algorithms. Thus, it can be concluded that the controller parameter values obtained from BSA-PI are best suited for feedback PI voltage controller design in all aspects. The hardware results are shown in Figure 15a,b. Table 7 shows the outcomes of the PEHS utilizing the BSA-PI controller, which are compared with current and other existing work in terms of voltage and switching frequency. Few authors have developed a boost converter for PEHS, but no one utilized optimization algorithms. The authors in [36,37,38] designed a boost converter to increase the output voltage for PEHS by varying the PI controller, power management circuit, and voltage regulator circuit, etc., without utilizing any optimization algorithm. In [36], the author designed a closed loop system, bridgeless boost rectifier utilizing a conventional PI controller with a 50 kHz switching frequency to increase the output voltage, and the model boosts 3.3 V DC with an input of 0.4 V AC. The author in [37] designed a DC-DC converter with a power management circuit with a 3 MHz switching frequency to increase the output voltage, and the model boosts 1.2 V DC with an input of 0.12 V AC. The author in [38] designed a boost converter from micro-energy sources with a 170 kHz switching frequency to maximize the output voltage, and the model boosts 3.3 V DC with an input of 0.25–0.4 V AC. Therefore, in this study, a BSA-PI controller is utilized to increase the output voltage of the PEHS. The advantage of the BSA-based PI voltage controller of the PEHS shows a great improvement, compared to the others techniques, with stability, boost maximum voltage, and faster response in terms of rise time and settling time, with changing loads, in simulation and experimental results. On the other hand, the disadvantage of the other methods is the required mathematical modeling and trial-and-error technique. Experimental results show that the output voltage is 6.06 V DC with an input of 0.3 V AC with a 10 kHZ switching frequency. From this table it is obvious that the proposed approach can obtain a higher optimal output voltage compared to the others. 

## 7. Conclusions

This paper presented an optimization approach to find the optimal parameter values of a PI voltage controller for a PEHS considering a specific vibration of a PVT for micro-energy harvesting. The proposed BSA-PI optimization technique is used to assist in avoiding the traditional trial-and-error procedure for finding the best values of Kp and Ki. To solve the optimization objective problem MAE, BSA was used, and the simulation and experimental results of this technique are compared with the results of the PSO to validate the outcomes. The obtained results clearly show that the BSA-PI controller outperforms the PSO-PI controller in terms of rise time, settling time. Finally, this prototype successfully boost the 0.3 V, with 65 Hz AC to 6.06 V DC. The output voltage is strongly regulated at 6.06 V through a closed-loop using a BSA-PI voltage controller that is suitable for micro-device applications. Naturally, vibration sources produce unstable excitation conditions (varying frequency and amplitude). In this regard the work might be extended for further experimentation in the future.

## Figures and Tables

**Table 1:** Piezoelectric vibration transducer parameters.

Parameter	PVT	Units
Piezo Material	5A4	E
Weight	10.4	grams
Stiffness	1.9 × 10^2^	N/m
Capacitance	232	nF
Rated Tip Deflection	±2.6	(mm_peak_)
Maximum Rated Frequency	52	Hz

**Table 2:** Equipment list for the experimental setup.

Equipment Name	Equipment Model	Equipment Quantity
PVT	EH220-A4-503YB	1
Amplifier Module	Smart material	1
Vibration Shaker	-	1
Digital Voltmeter	72-7130A	1
Copper-Board	-	2
Digital Oscilloscope	DSO × 2074A‎	1
dSPACE Controller	DS1104	1
Voltage Probe	-	2
Switch	-	5
Resistive load LED	-	5
Inductor L	3500 μH	1
Filter Capacitor C	300 μF	2
MOSFETs	SI9926CDY	1
Schottky Diodes	IN5819	5

**Table 3:** Analytical data from the PVT.

**Frequency (Hz)**	12	16	19	21	24	28	30	34	36	40	44	48	50	54	60	65	70	74
**Vrms**	0.3	0.5	0.8	0.9	1	1.2	1.7	2	1.26	1.17	1.2	1.27	1	1.3	2	2.75	0.5	0.2

**Table 4:** Values of output error by different techniques.

MAE	**Trial-and-Error**	**PSO**	**BSA**
	0.153716127	0.032125084	0.021602106

**Table 5:** Parameter settings of BSA-PI and PSO-PI.

Parameter	BSA-PI	PSO-PI
Population Size	50	50
Maximum Iteration	100	100
c1 and c2	-	2
E	2	-

**Table 6:** Comparison results obtained using BSA-PI and PSO-PI controllers.

Algorithms	Kp	Ki	Rise Time (Tr)	Settling Time (Ts)	Peak over Shoot (Po)	Steady State Error
BSA-PI	0.7	0.03	0.24 s	0.28 s	0%	0.1
PSO-PI	1	1.5	1.4 s	2.1 s	0%	0.1

**Table 7:** Comparison of the output voltage.

Component	[36]	[37]	[38]	This Work
Algorithm	N/A	N/A	N/A	BSA-PI
Vin (Input voltage)	0.4 V	0.12 V	(0.25–0.4) V	0.3 V
Vo (Output voltage)	3.3 V	1.2 V	3.3 V	6.06 V
Switching Frequency	50 kHz	3 MHz	170 kHz	10 kHz
Load	200 Ω	10 kΩ	30 kΩ	1.5 MΩ, 1 MΩ, 1000 kΩ, 500 kΩ, 300 kΩ
Application	Energy harvesting	Energy harvesting	Low power	Micro-devices

## References

[1] Peng S.W., Shih P.J., Dai C.L. (2015). Manufacturing and characterization of a thermoelectric energy harvester sing the CMOS-MEMS technology. Micromachines.

[2] Wang Y.J., Chen C.D., Lin C.C., Yu J.H. (2015). A nonlinear suspended energy harvester for a tire pressure monitoring system. Micromachines.

[3] Liu H., Chen T., Sun L., Lee C. (2015). An electromagnetic MEMS energy harvester array with multiple vibration modes. Micromachines.

[4] Hassanalieragh M., Soyata T., Nadeau A., Sharma G. (2016). UR-SolarCap: An open source intelligent auto-wakeup solar energy harvesting system for supercapacitor-based energy buffering. IEEE Access.

[5] Yang Z., Jean Z. (2016). Toward harvesting vibration energy from multiple directions by a nonlinear compressive-mode piezoelectric transducer. IEEE Trans. Mechatron..

[6] Mohamed R., Sarker M.R., Mohamed A. (2016). An optimization of rectangular shape piezoelectric energy harvesting cantilever beam for micro devices. Int. J. Appl. Electromagn. Mech..

[7] Rezaeisaray M., Gowini M., Sameoto D. (2015). Low frequency piezoelectric energy harvesting at multi vibration mode shapes. Sens. Actuators A Phys..

[8] Cui X., Teng M., Hu J. (2015). PSPICE-Based Analyses of the Vibration Energy Harvester System with Multiple Piezoelectric Units. Can. J. Electr. Comput. Eng..

[9] Ayaz M., Farjah E., Ghanbari T. A Novel Self-Starting Ultra Low-Power and Low-Voltage Two-Stage DC-DC Boost Converter for Microbial Energy Harvesting. Proceedings of the IEEE 4th International Conference on Consumer Electronics.

[10] Mahidur R.S., Ramizi M.A. (2014). Batteryless low input voltage micro-scale thermoelectric based energy harvesting interface circuit with 100mv start-up voltage. Przeglad Elektrotechniczny.

[11] Ramadass Y., Chandrakasan A. (2008). Minimum energy tracking loop with embedded DC/DC converter enabling ultra-low-voltage operation down to 250 mV in 65 nm CMOS. IEEE J. Solid-State Circuits.

[12] Ottman G.K., Hofmann H.F., Bhatt A.C., Lesieutre G.A. (2002). Adaptive piezoelectric energy harvesting circuit for wireless remote power supply. IEEE Trans. Power Electron..

[13] Ottman G.K., Hofmann H.F., Lesieutre G.A. (2003). Optimized piezoelectric energy harvesting circuit using step-down converter in discontinuous conduction mode. IEEE Trans. Power Electron..

[14] Antonio J.B., Barbi I. (2015). Input-series and output-series connected modular output capacitor full-bridge PWM DC-DC converter. IEEE Trans. Ind. Electron..

[15] Antonio J.B., Barbi I. Input-Series and Output-Series Connected Modular Full-Bridge PWM DC-DC Converter with Capacitive Output Filter and Common Duty Cycle. Proceedings of the 11th IEEE/IAS International Conference on Industry Applications (INDUSCON).

[16] Aldo B., Corsanini D., Landi A., Sani L. (2006). Circle based Criteria for performance evaluation of controlled DC/DC switching Converters. IEEE Trans. Ind. Electron..

[17] Basilio J.C., Matos S.R. (2002). Design of PI and PID controllers with transient performance specification. IEEE Trans. Educ..

[18] LE T., Sentieys O., Berder O., Pegatoquet A. Power Manager with PID Controller in Energy Harvesting Wireless Sensor Networks. Proceedings of the IEEE International Conference on Green Computing and Communications.

[19] Amala J., Rajan S., Vengatesh R. Design and analysis of high frequency Soft-Switching Boost Converter employing Electronic PI-Controller. Proceedings of the International Conference on Emerging Trends in Electrical and Computer Technology (ICETECT).

[20] Hazzab A., Bousserhane I.K., Zerbo M., Sicard P., Murphy G.V. (2006). Real time implementation of fuzzy gain scheduling of PI controller for induction motor machine control. Neural Process. Lett..

[21] Ngo P.D., Shin Y.C. (2015). Gain estimation of nonlinear dynamic systems modeled by an FBFN and the maximum output scaling factor of a self-tuning PI fuzzy controller. Eng. Appl. Artif. Intell..

[22] Perry A.G., Feng G., Liu Y.F. (2007). A design method for PI-like fuzzy logic controller for DC-DC converter. IEEE Trans. Ind. Electron..

[23] Elmas C., Yigit T. (2007). Genetic algorithm based on-line tuning of a PI controller for a switched reluctance motor drive. Electr. Power Compon. Syst..

[24] Mo H., Yin Y. Research on PID Tuning of Servo-System Based on Bacterial Foraging Algorithm. Proceedings of the 7th International Conference on Natural Computation (ICNC).

[25] Afrozi M.N., Aghdam M.H., Naebi A., Aghdam S.H. Simulation and Optimization of Asynchronous AC Motor Control by Partial Swarm Optimization (PSO) and Emperor Algorithm. Proceedings of the 5th UKSim European Symposium on Computer Modeling and Simulation (EMS).

[26] Jamal A., Hannan M.A., Mohamed A., Abdolrasol M. (2016). Fuzzy logic speed controller optimization approach for induction motor drive using backtracking search algorithm. Measurement.

[27] Civicioglu P. (2013). Backtracking search optimization algorithm for numerical optimization problems. Appl. Math. Comput..

[28] Astrom K.J., Hagglund T. (1988). Automatic Tuning of PID Controllers.

[29] Youssef O., Kamal A.H., Luc A.G. (2011). Packed U cells multilevel converter topology: Theoretical study and experimental validation. IEEE Trans. Ind. Electron..

[30] dSPACE DS1104 (2008). Hardware Installation and Configuration and ControlDesk Experiment Guide.

[31] Zainal S., Toh L.S., Mohd Z.R. Hardware Implementation of the High Frequency Link Inverter Using dSPACE DS1104 Digital Signal Processing Board. Proceedings of the IEEE International Power and Energy Conference (PECon’06).

[32] Lin Q., Gao L., Li X., Zhang C. (2015). A hybrid backtracking search algorithm for permutation flow-shop scheduling problem. Comput. Ind. Eng..

[33] Song X., Zhang X., Zhao S., Li L. (2015). Backtracking search algorithm for effective and efficient surface wave analysis. Comput. Ind. Eng..

[34] Askarzadeh A., Coelho L.S. (2014). A backtracking search algorithm combined with Burger’s chaotic map for parameter estimation of PEMFC electrochemical model. Int. J. Hydrog. Energy.

[35] El-Fergany A., Coelho L.S. (2015). Optimal allocation of multi-type distributed generators using backtracking search optimization algorithm. Int. J. Electr. Power Energy Syst..

[36] Wang H., Tang Y., Khaligh A. (2013). A Bridgeless Boost rectifier for low-voltage energy harvesting applications. IEEE Trans. Power Electron..

[37] Richelli A., Comensoli S., Kovacs-Vajna Z.M. (2012). A DC/DC boosting technique and power management for ultralow-voltage energy harvesting applications. IEEE Trans. Ind. Electron..

[38] Bertacchini A., Scorcioni S., Cori M., Larcher L., Pavan P. 250 mv Input Boost Converter for Low Power Applications. Proceedings of the IEEE International Symposium on Industrial Electronics (ISIE).

